# Longitudinal Profiling of the Human Milk Microbiome from Birth to 12 Months Reveals Overall Stability and Selective Taxa-Level Variation

**DOI:** 10.3390/microorganisms13081830

**Published:** 2025-08-05

**Authors:** Ruomei Xu, Zoya Gridneva, Matthew S. Payne, Mark P. Nicol, Ali S. Cheema, Donna T. Geddes, Lisa F. Stinson

**Affiliations:** 1 School of Molecular Sciences, The University of Western Australia, Crawley, WA 6009, Australia; 2 UWA Center for Human Lactation Research and Translation, Crawley, WA 6009, Australia; 3 ABREAST Network, Perth, WA 6000, Australia; 4 Division of Obstetrics and Gynaecology, School of Medicine, The University of Western Australia, Crawley, WA 6009, Australia; 5 School of Biomedical Sciences, The University of Western Australia, Crawley, WA 6009, Australia; 6 The Kids Research Institute Australia, Nedlands, WA 6009, Australia

**Keywords:** human milk microbiome, determinants, breastfeeding, 16S rRNA gene sequencing

## Abstract

Human milk bacteria contribute to gut microbiome establishment in breastfed infants. Although breastfeeding is recommended throughout infancy, temporal variation in the milk microbiome—particularly beyond solid food introduction—remains understudied. We analyzed 539 milk samples from 83 mother–infant dyads between 1 week and 12 months postpartum using full-length 16S rRNA gene sequencing. The microbiota was dominated by *Streptococcus* (34%), *Cutibacterium* (12%), and *Staphylococcus* (9%), with marked inter-individual variation. Microbiome profiles remained largely stable across lactation, with only six taxa showing temporal fluctuations, including increases in typical oral bacteria such as *Streptococcus salivarius*, *Streptococcus lactarius*, *Rothia mucilaginosa*, and *Granulicatella adiacens*. Richness and evenness were higher at 1 week compared to 1 month postpartum (*p* = 0.00003 and *p* = 0.007, respectively), then stabilized. Beta diversity also remained stable over time. Maternal pre-pregnancy BMI was positively associated with *Gemella haemolysans* (*p* = 0.016), while *Haemophilus parainfluenzae* was more abundant in milk from mothers with allergies (*p* = 0.003) and those who gave birth in autumn or winter (*p* = 0.006). The introduction of solid food was linked to minor taxonomic shifts. Overall, the milk microbiome remained robustly stable over the first year of lactation, with limited but notable fluctuations in specific taxa. This study supports the role of human milk as a consistent microbial source for infants and identifies maternal BMI, allergy status, and birth season as key variables warranting further investigation.

## 1. Introduction

It is estimated that a breastfed infant ingests approximately 8 × 10^6^ bacteria from human milk every day [1], and that these bacteria contribute to the establishment of the infant oral and gut microbiome [2,3,4,5,6,7,8]. The milk microbiome is made up predominantly of typical skin and oral bacteria including streptococci and staphylococci [9], which are postulated to originate from the infant oral cavity, maternal skin, or maternal gut [10,11]. Although the World Health Organization recommends that young children continue to receive human milk in addition to solid foods after six months of age [12], there remains a lack of evidence regarding longitudinal changes in the milk microbiome over the lactation period. Previously published longitudinal milk microbiome studies are largely restricted to the first few months of lactation. While some studies have reported that the milk microbiome is stable over time [13,14,15], others have reported variance in diversity and composition [16,17]. However, of these studies, only one has sampled beyond the first six months postpartum. This represents a key limitation of the current literature, as the introduction of solid foods around this time may influence the milk microbiome indirectly via changes to the infant oral microbiome [18]; however, this hypothesis has not previously been tested.

Diversity within the milk microbiome may influence the breadth and resilience of microbial seeding in the infant gut and oral cavity. A more diverse microbial community may support broader colonization potential and more effective immune system training during early life, though this remains an area of active investigation. As such, understanding patterns of milk microbiome diversity across lactation may have important implications for infant health.

Numerous maternal, infant, and environmental factors have been identified as potential determinants of the human milk microbiome, including mode of delivery [17,19], mode of feeding [20,21], and maternal pre-pregnancy body mass index (BMI) [19,22]. However, there is a lack of consistency in these types of findings between studies. These inconsistencies may be attributed to differences in study populations [23,24], geographical locations [25], maternal diets [26,27], sample sizes, or analytical approaches. Other sample-specific factors, including milk collection methods (hand or pump, aseptic or non-aseptic), may contribute to differences seen between different studies [20,28,29].

Due to limited consensus on the factors influencing the composition of the human milk microbiome, and the scarcity of longitudinal data beyond the early postpartum period, this study aimed to characterize the temporal development of the milk microbiome over the first year postpartum in healthy Australian mother–infant dyads. We also sought to evaluate whether specific maternal and infant characteristics, including maternal pre-pregnancy BMI, allergy status, birth season, and timing of solid food introduction, were associated with variation in microbial composition or diversity. We hypothesized that certain taxa may show temporal shifts, particularly around the introduction of solid foods, and that specific maternal or infant variables could influence microbial profiles.

## 2. Materials and Methods

### 2.1. Study Design and Sample Collection

This study utilized samples and data from 84 mother–infant dyads (singletons only) from the BLOSOM (Breastfeeding Longitudinal Observational Study of Mothers and kids) cohort [30]. Mothers who intended to exclusively breastfeed prior to the introduction of solid foods and have a duration of breastfeeding of at least 12 months were invited to participate. Exclusion criteria included the following: major pregnancy complications, maternal smoking, preterm birth, and congenital diseases. This study was approved by the Human Research Ethics Committee of the University of Western Australia (RA/4/20/4023), and all mothers provided informed written consent.

As previously described [30], human milk samples were collected at 2–6 days and at 1, 2, 3, 4, 5, 6, 9, and 12 months postpartum. All samples were collected via a single hand-expression event (not pooled over 24 h) from one breast selected by the mother for consistency across time points. Mothers were instructed to refrain from breastfeeding or expressing from the selected breast for at least two hours prior to collection. Aseptic sample collection technique included thorough handwashing, use of disposable gloves, and cleaning of the nipple and areola with prep pads containing 70% isopropyl alcohol and 2% chlorhexidine digluconate (Reynard Health Supplies, Sydney, Australia), followed by a rinse with sterile saline and drying with sterile gauze. Up to 20 mL (or as much as possible) of milk was hand-expressed directly into 50 mL certified sterile Sarstedt polypropylene tubes. Samples were stored in the participants’ home fridge at 4 °C for up to 18 h before being transported to the laboratory, where they were stored at −80 °C until further analysis.

### 2.2. DNA Extraction and Amplification

Total DNA was extracted from 1 mL aliquots of each milk sample using the QIAGEN MagAttract Microbial DNA Isolation Kit (QIAGEN, Hilden, Germany) on the Kingfisher Flex platform following the manufacturer’s instructions. A blank (reagents only) negative extraction control was included in each extraction batch.

Amplification of the full-length 16S rRNA gene was performed in 30 µL reactions consisting of 1× AccuStart II PCR ToughMix (Quantabio, Beverly, MA, USA), 3 µL nuclease-free water, 0.3 µM each of the PacBio barcoded forward (27F) and reverse (1492R) primers (Integrated DNA Technologies, Melbourne, Australia), 0.75 µL each of dsDNase and DTT (ArcticZymes PCR decontamination kit, ArcticZymes Technology, Tromsø, Norway), and 6 µL of template. PCR reagents (including primers) were treated using the ArcticZymes PCR Decontamination kit prior to the addition of template. The PCR cycling conditions consisted of an initial heating stage of 94 °C for 3 min, followed by 38 cycles of 94 °C for 30 s, 52 °C for 30 s, and 72 °C for 2 min 30 s, as well as a final extension stage of 72 °C for 5 min. A negative template amplification control was included in each PCR plate to control for PCR reagent-derived contamination. Amplicon size and concentration were assessed using a QIAXcel capillary gel electrophoresis system (QIAGEN, Germany). Amplicons were normalized and pooled in equimolar concentrations, prior to purification and concentration using Macherey Nagel NucleoMag NGS beads (Macherey Nagel, Düren, Germany).

### 2.3. PacBio Sequencing

Sequencing was carried out using the PacBio Sequel II at the Australian Genome Research Facility. Each pool was sequenced across one SMRT cell. Sequencing data were processed using PacBio SMRTLink to generate demultiplexed FASTQ files.

#### Sequence Processing

FASTQ files were processed using mothur v.1.48.0 [31]. Quality filtering was performed based on amplicon length (1336–1742 bp), number of homopolymers (<9), and alignment (position 1044–43116). Alignment was performed using the SILVA reference alignment v132 [32]. Chimeric sequences were removed using VSEARCH [33], and operational taxonomic units (OTUs) were created using the cluster split method with a cutoff distance of 0.03.

Subsampling was performed to 1100 reads, resulting in an average coverage of 96%. Our subsampling excluded 48 low-yield samples from further analysis, including all samples from one participant, reducing the participant number to 83. The remaining samples had average sequence read counts of 13,154 ± 7858 and a median of 11,054. Alpha diversity (Shannon diversity and richness) and beta diversity (Bray–Curtis distances) were generated from subsampled data. Bray–Curtis dissimilarity was used to assess beta diversity, as it captures abundance-driven differences in community composition and is well suited for detecting temporal shifts and associations with metadata. Center-log ratio (CLR) transformation was performed on OTU count data prior to differential abundance analysis. OTUs with a mean relative abundance of ≥0.5% were included in the analysis. Initial genus-level taxonomic assignments for each OTU were obtained using the SILVA taxonomy database v132, followed by species-level assignments manually mapped using BLAST v2.17.0 (sequence identity cutoff of ≥98.5%) [34]. In cases where there was an equally good match for more than one species, genus-level identity is provided. Taxonomic assignments for sequences recovered from the negative extraction and negative amplification controls are provided in Table S1.

### 2.4. Statistical Analysis

All statistical analyzes and data visualization were performed in R (version 4.4.3) [35]. To analyze longitudinal changes in the milk microbiome and to assess its relationships with microbiome covariates, multivariable linear mixed-effects models were fitted for each microbiota feature (Shannon diversity, OTU-level richness, and CLR-transformed abundance of each OTU) using the R package lme4 v1.1-37. Fixed effects included the following: time (with 1 month as the reference value), maternal age at delivery, maternal allergy (self-reported), parity (categorical: 1, 2, ≥3), pre-pregnancy BMI (underweight/normal weight or overweight/obese), delivery mode (vaginal or a cesarean section), birth season (spring/summer or autumn/winter), intrapartum antibiotic prophylaxis, infant sex, and the presence of furry pets (cats/dogs) in the home. Time was included as an interaction term for each variable, and participant ID was included as a random effect. Due to low numbers of mothers with underweight (*n* = 3) or obese (*n* = 7) pre-pregnancy BMI, this variable was grouped into two categories (underweight/normal weight, overweight/obese). Similarly, due to low numbers of each category of cesarean section (pre-labor *n* = 16, intrapartum *n* = 10), all cesarean deliveries were grouped together. Additionally, there were only 7 infants who were born in summer, while the number of infants born in spring, autumn, and winter was 28, 23, and 25, respectively. Therefore, the birth season was grouped into two categories (spring/summer or autumn/winter). Benjamini–Hochberg correction for multiple comparisons was used. Factors associated with beta diversity of the milk microbiome were identified in backward selection models using the adonis2 function of the vegan R package v2.7-1 [36]. Microbial volatility analysis was performed to assess the degree of change in Bray–Curtis distance over time, as described by Bastiaanssen et al. [37].

To investigate changes in the milk microbiome associated with solid food introduction, 75 mother–infant pairs with data on solid food introduction were selected. Samples collected one time point before the introduction of solid food were classified as “pre-solids” (range: 3–6 months), and samples collected one time point after solid food introduction were classified as “post-solids (1)” (range: 4–9 months). Additionally, in acknowledgment of the fact that “post-solids (1)” samples may have been collected as little as one day after introduction of solids, and that the impact of solid food introduction on the milk microbiome may be lagged, we included an additional time point in the analysis—these samples were collected two time points after introduction of solids and were classified as “post-solids (2)” (range: 5–12 months). The impacts of solid food exposure on alpha diversity measures and CLR-transformed OTU abundances were assessed using Kruskal–Wallis rank sum tests with pairwise Wilcoxon tests. The impact of solid food exposure on milk beta diversity was assessed using PERMANOVA with subsequent pairwise PERMANOVA using the adonis2 function of the vegan package v2.7-1.

A *p*-value < 0.05 was considered significant for all statistical analyses. Benjamini–Hochberg corrected *p*-values are reported throughout the text, and uncorrected *p*-values are reported in Supplementary Table S2.

## 3. Results

A total of 539 human milk samples from 83 mother–infant dyads were included in the analysis. The majority of participants were Caucasian and multiparous (Table 1). All mothers exclusively or predominantly breastfed their infants, and all infants were still breastfed at 9 months, with only 4 having ceased breastfeeding by 12 months.

**Figure 1:**
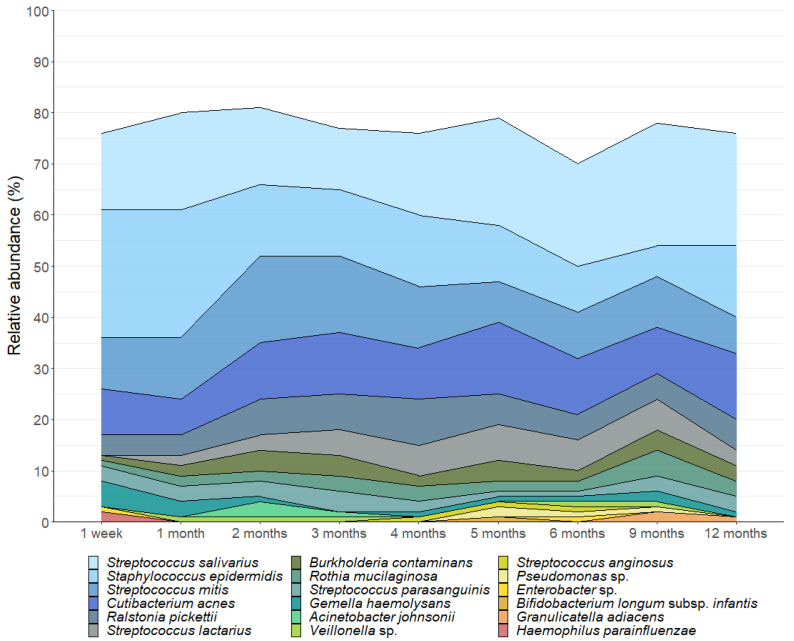
Composition of the human milk microbiota in the first 12 months postpartum. OTUs with a mean relative abundance of ≥0.5% are displayed.

**Figure 2:**
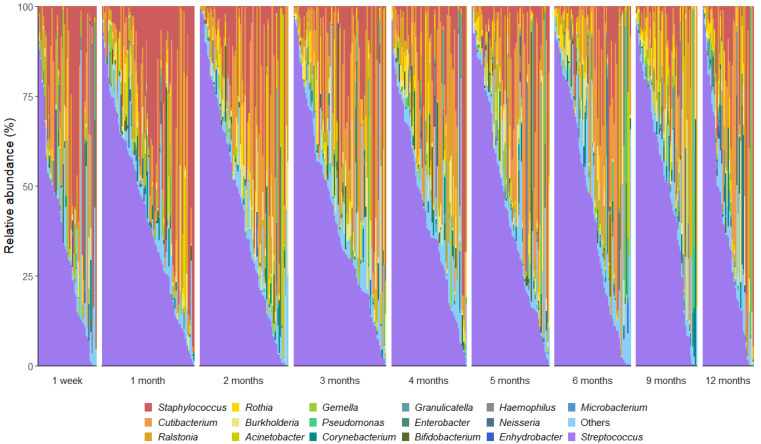
Genus-level interindividual variation in human milk microbiota profiles across the first 12 months postpartum. Genera that made up <2% mean relative abundance are grouped together as “others”.

We detected 520 bacterial genera and 17,954 OTUs in this sample set. A total of 18 OTUs with an average relative abundance ≥ 0.5% were identified, representing 85% of the total bacterial sequences recovered from the milk samples. As reported in other cohorts [13,21,38], the milk microbiota was dominated by *Streptococcus* (mean relative abundance 34%), *Cutibacterium* (12%), and *Staphylococcus* (9%) (Figure 1). Notably, the important infant gut commensal *Bifidobacterium* was present in 33% (180 out of 539) of the milk samples, with 68 mothers (82%) having at least one sample containing this genus. *Bifidobacterium* made up 1% of the bacterial DNA profiles with *Bifidobacterium longum* subsp. *infantis* being the most dominant species. However, there was a high level of inter-individual variation in the human milk microbiota profiles (Figure 2).

### 3.1. The Milk Microbiome Was Largely Stable over Time

**Figure 3:**
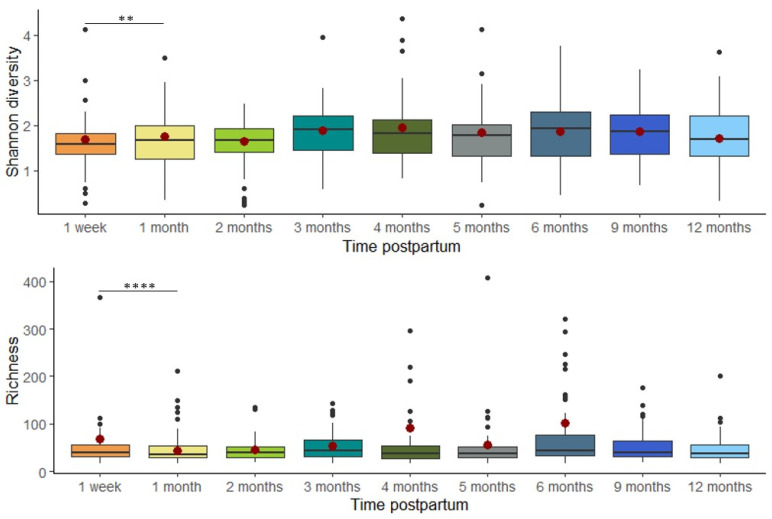
Alpha diversity was significantly elevated at 1 week compared to 1 month postpartum. The box plots represent Shannon diversity/richness at each time point. The model-predicted means at each time point are represented by red dots. The model-predicted means for richness are not estimable at 9 and 12 months due to insufficient data (** *p* < 0.01, **** *p* < 0.0001).

**Figure 4:**
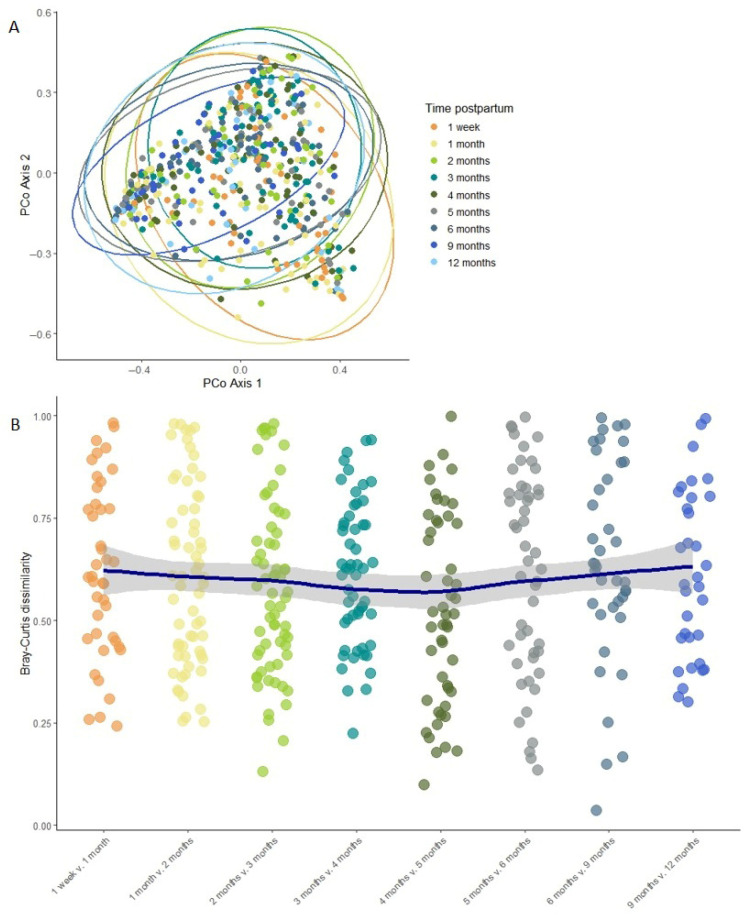
Milk microbiome beta diversity remains largely stable over time. (**A**) PCoA plot of Bray–Curtis distances between different time points. The ellipses show the 95% confidence level. (**B**) Volatility of the milk microbiome between subsequent time point pairs. Each point represents one mother’s dissimilarity between two consecutive time points; the LOESS line shows overall trend with the shaded area representing the 95% confidence intervals.

Overall, milk bacterial diversity was largely stable across the study period, apart from elevated Shannon diversity (estimate = 2.976, *p* = 0.007) and richness (estimate = 260.749, *p* = 0.00003) observed at 1 week compared to 1 month postpartum, based on linear mixed effects modeling (Figure 3). Beta diversity remained stable over time, with no significant differences in overall community composition between time points as assessed by PERMANOVA (Bray–Curtis dissimilarity; *R*^2^ = 0.007, *p* = 0.997; Figure 4A). To further assess individual-level compositional stability over time, we calculated Bray–Curtis dissimilarity between consecutive time points for each participant. These pairwise dissimilarity values (e.g., between week 1 and month 1, month 1 and month 2, etc.) varied widely across individuals (ranging from 0.003 to 0.999) but did not show any consistent trend over time. A LOESS curve fitted to the dissimilarity data confirmed that volatility remained stable throughout the study period (Figure 4B), further supporting the overall temporal stability of the milk microbiome.

**Figure 5:**
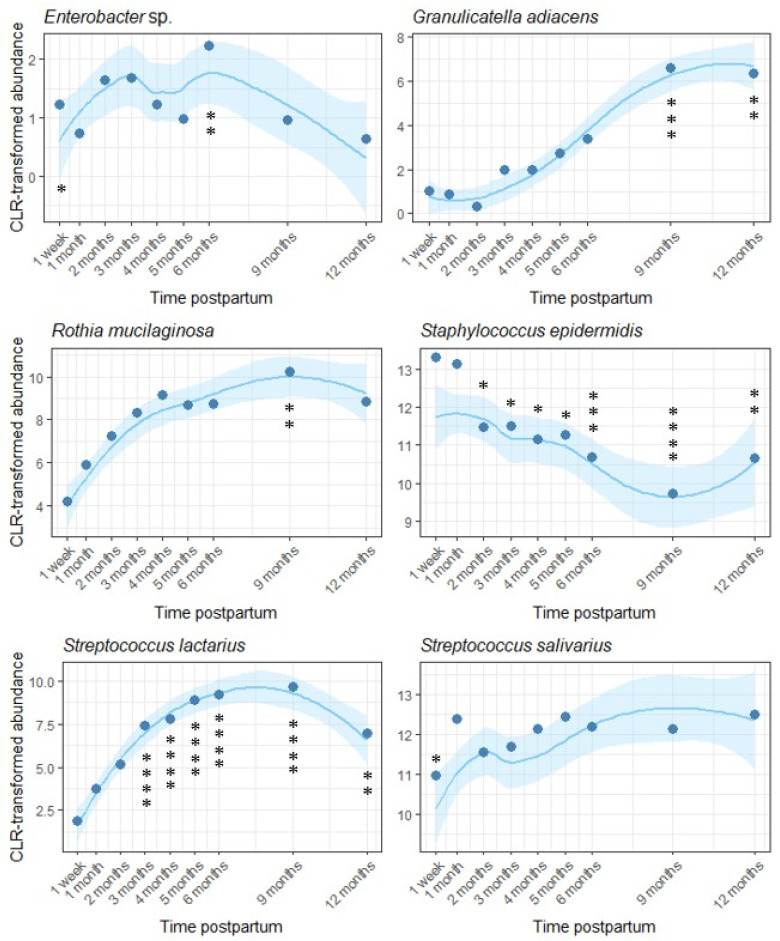
Temporal variation in six OTUs within the human milk microbiota across the first year of lactation. One month postpartum was used as the reference level. Loess lines represent CLR transformed abundance at each time point, and the shaded areas represent the 95% confidence intervals. Model-predicted means at each time point are represented by dots (* *p* < 0.05, ** *p* < 0.01, *** *p* < 0.001, **** *p* < 0.0001).

Of the 18 OTUs analyzed here, we detected fluctuations over time in the relative abundance of six (Figure 5). Of these, two differed at one time point only (*Rothia mucilaginosa*, elevated at 9 months, *p* = 0.007; *Streptococcus salivarius*, reduced at 1 week, *p* = 0.036), while four (*Staphylococcus epidermidis*, *Streptococcus lactarius*, *Enterobacter* sp., and *Granulicatella adiacens*) underwent multiple changes over time. The relative abundance of *S. epidermidis* decreased over the course of lactation, while the relative abundance of *Enterobacter* sp., *G. adiacens*, and *S. lactarius* increased.

### 3.2. Determinants of the Human Milk Microbiota

**Figure 6:**
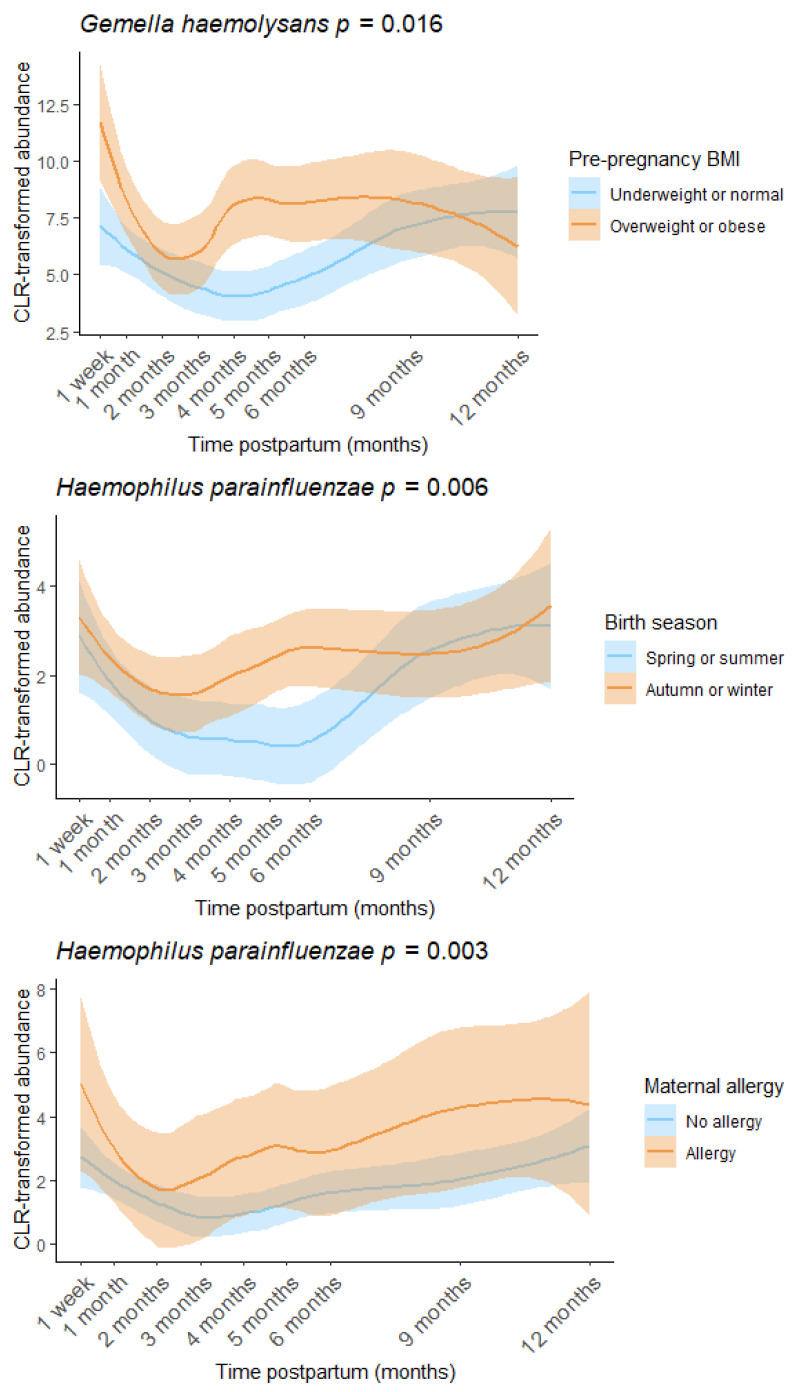
Pre-pregnancy BMI, birth season, and maternal allergy were associated with the human milk microbiome. Loess lines represent CLR transformed abundance at each time point, and the shaded areas represent the 95% confidence intervals.

Pre-pregnancy BMI, birth season, and maternal allergy were found to be associated with individual human milk OTUs (Figure 6, Table S2). Other factors investigated, including delivery mode, parity, and infant sex, were not significantly associated with milk microbiome composition or diversity in this cohort. There was a positive association between pre-pregnancy BMI and the relative abundance of *Gemella haemolysans* (estimate = 2.250, *p* = 0.016), while both maternal allergy and autumn/winter birth were associated with increased *Haemophilus parainfluenzae* (allergy: estimate = 1.976, *p* = 0.003, season: estimate = 1.372, *p* = 0.006). Maternal age was weakly associated with increased Shannon diversity (estimate = 0.043, *p* = 0.044).

### 3.3. Introduction of Solid Foods Is Associated with Minor Changes in the Milk Microbiota

**Figure 7:**
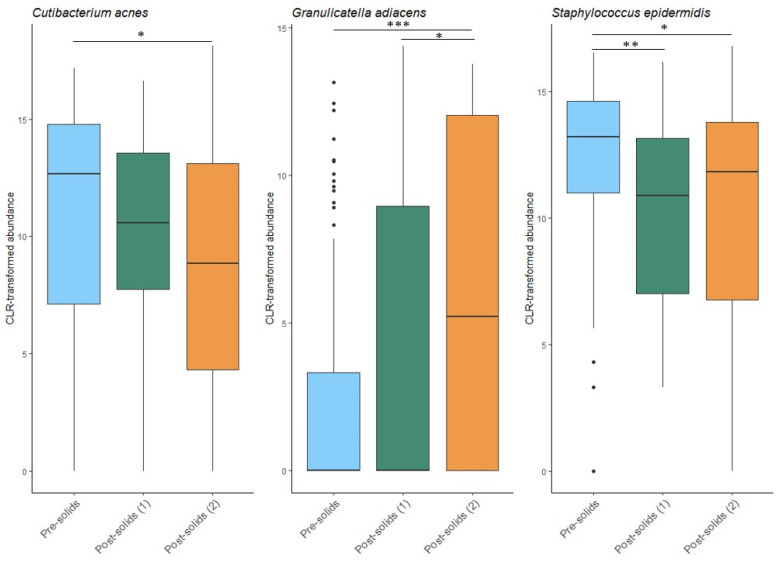
Impact of solid food introduction on the human milk microbiota. Pre-solids: the sample collected immediately prior to the first introduction of solid foods. Post-solids (1): the first sample collected after the introduction of solid foods. Post-solids (2): the second sample collected at two sample time points after the introduction of solid foods. * *p* < 0.05, ** *p* < 0.01, *** *p* < 0.001.

The introduction of solid food to the infant’s diet was not associated with Shannon diversity (*p* = 0.473), richness (*p* = 0.218), or beta diversity (*p* = 0.141) of the human milk microbiome. However, of the 18 OTUs analyzed here, the relative abundance of three (*S. epidermidis*, *Cutibacterium acnes*, and *G. adiacens*) significantly changed after solid food introduction (Figure 7). Both *C. acnes* and *S. epidermidis* decreased after solid food introduction. The relative abundance of *S. epidermidis* was significantly lower immediately following (*p* = 0.006) and two time points following solid food introduction (*p* = 0.040), while the relative abundance of *C. acnes* did not decrease until two time points following solid food introduction (*p* = 0.012). A similar delayed impact was observed for *G. adiacens,* with the increase in this bacterial species not apparent until the second time point, after the infants began eating solid foods (*p* = 0.0006).

## 4. Discussion

This study is one of the few longitudinal studies investigating the temporal development of the human milk microbiome beyond the first few months of lactation. In line with previous studies [13,14], we found that the human milk microbiome was largely stable over the first year. The heightened microbial diversity observed during the first week of lactation may represent an initial period of community adaptation to the compositional shift from colostrum to mature milk. After this initial transition, milk diversity remained stable. The composition of the milk microbiome in the BLOSOM cohort was highly similar to that reported in other geographically dissimilar cohorts [16,21,24,39], with typical skin taxa (e.g., *Staphylococcus* and *Cutibacterium*) as well as typical oral/respiratory taxa (e.g., *Streptococcus* and *Rothia*) predominant. The robust stability of human milk composition over time and between populations demonstrates a tightly regulated biological system that supports infant microbial exposure across lactation.

As an important infant gut genus and one of the earliest colonizers in the infant gut, *Bifidobacterium* has been shown to be vertically transmitted from mothers to infants via milk [2,3,4,6,7], and its growth is promoted by human milk components, such as human milk oligosaccharides [40,41,42]. This genus made up 1% of the bacterial DNA profiles in our samples and was detected in 33% of the samples. The proportion of human milk samples containing *Bifidobacterium* in this cohort was similar to that reported previously (35%) [43]. However, a decrease in infant gut *Bifidobacterium* has been observed in some developed countries over the past decades [25,44,45,46], highlighting the need for targeted analysis of human milk *Bifidobacterium* to support potential interventions to promote healthy infant colonization.

Of the maternal and infant characteristics investigated here, birth season, maternal allergy, pre-pregnancy BMI, and maternal age were identified as potential determinants of the human milk microbiome. We observed an increased abundance of *H. parainfluenzae*, a common oral and respiratory tract commensal, in allergic mothers and those who gave birth in autumn or winter. The seasonal association is likely driven by increased circulation of *Haemophilus* in colder months, when respiratory carriage rates tend to rise [47,48]. Infants born during autumn and winter may therefore be more likely to harbor this species in their oral or upper respiratory tract, leading to increased transfer to the mammary gland via a retrograde flow of milk [49]. The association with maternal allergy may reflect previous findings linking *H. parainfluenzae* to atopic manifestations [50], suggesting that allergic mothers could carry higher levels of this species themselves, contributing to its increased presence in milk. A previous study of atopic lactating women reported that both birth season and type of atopic condition (food allergy, allergic rhinitis, asthma, eczema) were associated with distinct shifts in milk microbiome composition [51], supporting our findings.

A higher pre-pregnancy BMI was associated with an increased relative abundance of *G. haemolysans* in milk. This finding aligns with previous research reporting higher *Gemella* abundance in milk from mothers with an overweight pre-pregnancy BMI, gestational diabetes, and impaired glucose tolerance [23], as well as in the gut microbiome of non-lactating individuals with obesity or diabetes [52,53]. Although the underlying mechanisms remain unclear, some evidence suggests a positive association between *Gemella* abundance and intake of high-glycemic-index foods, which may promote inflammatory immune responses [54]. While we found no differences in milk microbiome diversity by pre-pregnancy BMI in our cohort, other studies have reported lower alpha diversity (e.g., Shannon index) in milk from mothers with an overweight pre-pregnancy BMI [19,55]. Further studies with more mothers in each BMI category are required to better characterize the relationship.

We observed a weak positive association between maternal age and Shannon diversity of the human milk microbiome. One hypothesis to explain this finding is that older mothers may have breastfed previous children, with exposure to other infant oral microbiota increasing milk microbial diversity. However, while maternal age and parity were correlated in our study (Kendall’s τ = 0.125, *p* = 0.0002), parity itself was not associated with milk microbiome diversity in multivariate models. This suggests that prior lactation exposure does not explain the observed relationship. Although microbial communities across the body are known to change with age [56], the relatively narrow age range in our cohort (32.9 ± 4.5 years) limits the likelihood of age-related shifts being biologically meaningful here. Given the modest effect size and weak statistical significance, this association should be interpreted cautiously and warrants further investigation in larger, more age-diverse cohorts.

Although mode of delivery is frequently cited as a factor influencing the infant gut microbiome [57], its impact on the human milk microbiome remains uncertain. In the present study, mode of delivery was not associated with any differences in milk microbiome composition or diversity, consistent with prior longitudinal studies reporting minimal or no effect of birth mode on the milk microbiome [17,21]. This suggests that differences in the infant gut microbiome following cesarean delivery cannot be explained by differences in the milk microbiome of mothers delivering via cesarean compared to those delivering vaginally.

Although we anticipated that the introduction of solid foods might substantially alter the milk microbiome, the overall impact was limited. Of the 18 OTUs analyzed here, only 3 showed significant changes: *C. acnes* and *S. epidermidis* decreased, while *G. adiacens* increased. No changes in alpha or beta diversity were observed. This was somewhat unexpected given previously published findings from this very cohort, in which the infant oral microbiome underwent significant compositional and diversity shifts following the introduction of solid food [18]. Given the frequent contact between the infant oral cavity and the breast during breastfeeding, we anticipated that changes in the infant oral microbiome might be reflected in the milk. However, the milk microbiome appeared largely resistant to dietary transition. The few changes observed, particularly in skin-associated taxa such as *S. epidermidis* and *C. acnes*, may instead reflect behavioral changes, such as reduced breastfeeding frequency and decreased maternal–infant skin contact, rather than direct microbial transfer from the infant oral cavity.

This study has several key strengths. Compared to many previous investigations in the field, it includes a relatively large sample size and a longer follow-up period, enabling more detailed temporal characterization. By extending beyond the first few months of lactation, our findings help to address critical gaps in understanding how the milk microbiome evolves over time, particularly beyond the introduction of solid foods. The use of full-length 16S rRNA gene sequencing further strengthens our approach, allowing species-level taxonomic resolution. However, several limitations should be acknowledged. Although our cohort size was relatively modest compared to some cross-sectional studies, the dense longitudinal sampling across multiple time points strengthens our ability to examine temporal trends within individuals—a key gap in the existing literature. The cohort was mostly Caucasian (88%) and multiparous (77%), which may limit generalization of findings. Maternal allergy status was self-reported, and therefore subject to potential misclassification bias. Additionally, we were unable to assess certain factors that may influence the human milk microbiome, such as breast pump use and pump cleaning practices, maternal diet, exercise, or medication and supplement use. As these factors may significantly influence the milk microbiome, future longitudinal studies incorporating these variables will be valuable to further elucidate their roles in shaping microbial composition and diversity. Finally, while our analyses identified associations between the milk microbiome and maternal characteristics (allergy status, BMI), the number of participants in some subgroups was relatively small, and groups were not evenly distributed. Although we employed statistical approaches suitable for unbalanced data, larger studies with targeted recruitment will be important to validate and expand on these observations.

Overall, this study demonstrates that the human milk microbiome remains remarkably stable across the first year of lactation. While fluctuations were observed in six bacterial species, four of which had a moderate relative abundance of <5%, the broader community structure and alpha diversity showed minimal change over time. Factors such as pre-pregnancy BMI, season of birth, and maternal allergy were associated with shifts in the relative abundance of specific taxa, yet these associations did not substantially alter the overall microbial landscape. Similarly, the introduction of solid food—a major dietary milestone—was not associated with changes in milk microbial diversity, and only three individual taxa were affected. These findings suggest that, despite dynamic changes occurring in both mother and infant during the first year of life, the human milk microbiome maintains a high degree of stability. This consistency may serve an important biological role, providing infants with a reliable source of commensal and potentially beneficial microbes during a critical period of immune and metabolic development. Importantly, the stability of the milk microbiome even after the introduction of solids reinforces the value of continued breastfeeding throughout infancy. These results contribute to a growing body of work highlighting the resilience of the milk microbiome and underscore the need for future research focused on the functional roles and implications of the milk microbiome for maternal and infant health.

In summary, the human milk microbiome exhibits remarkable stability throughout the first year postpartum, with only minor fluctuations in specific bacterial taxa, highlighting its resilience and potential role as a consistent microbial source for infants.

## Figures and Tables

**Table 1:** Participant characteristics (*n* = 83).

Maternal Characteristics	Mean ± SD or *n* (%)
Age at delivery (years)	32.9 ± 4.5
Maternal ethnicity: Caucasian	73 (88.0)
Maternal ethnicity: Other	10 (12)
Under or normal weight pre-pregnancy BMI *	44 (72.1)
Overweight or obese pre-pregnancy BMI *	17 (27.9)
Parity: 1	19 (22.9)
Parity: 2	38 (45.8)
Parity: >2	26 (31.3)
Vaginal delivery	57 (68.7)
Cesarean delivery	26 (31.3)
Intrapartum antibiotic prophylaxis *	35 (44.3)
Maternal allergy *	15 (18.5)
Pets (cats and/or dogs) *	41 (64.1)
Infant Characteristics	Mean ± SD or *n* (%)
Birth gestation (weeks)	39.3 ± 1.1
Introduction of solid food (weeks)	23.3 ± 3.9
Female	44 (53.0)
Spring or summer birth	35 (42.2)
Autumn or winter birth	35 (42.2)
Formula use at 1 week #	4 (4.8)
Formula use at 1 month #	2 (2.4)
Formula use at 2 months #	2 (2.4)
Formula use at 3 months #	6 (7.2)
Formula use at 4 months #	3 (3.6)
Formula use at 5 months #	5 (6.0)
Formula use at 6 months #	12 (14.5)
Formula use at 9 months #	8 (9.6)
Formula use at 12 months #	4 (4.8)

* Incomplete records. BMI: 22 missing records. Intrapartum antibiotic prophylaxis: 4 missing records. Allergy: 2 missing records. Pets: 19 missing records. Note: # refers to use in the period between this sample collection and the previous sample collection.

## Data Availability

Raw sequence read data are available at the NCBI SRA (BioProject accession: PRJNA1229851).

## Supplementary Materials

The following supporting information can be downloaded at: https://www.mdpi.com/article/10.3390/microorganisms13081830/s1. Table S1: Genera detected from negative extraction (EC) and negative amplification (NTC) controls; Table S2: Outputs of multivariate linear mixed effects models for the impact of maternal and infant characteristics on milk microbiota alpha diversity and composition.

## Abbreviations

The following abbreviations are used in this manuscript:

BMI	Body mass index
DNA	Deoxyribonucleic acid
OTU	Operational taxonomic unit
rRNA	Ribosomal ribonucleic acid
