# Supplementary material for: Longitudinal Profiling of the Human Milk Microbiome from Birth to 12 Months Reveals Overall Stability and Selective Taxa-Level Variation
Source: Microorganisms. 2025 Aug 5;13(8):1830. doi: 10.3390/microorganisms13081830 (PMC12388394; doi:10.3390/microorganisms13081830)
Supplement: Supplementary file 1 [file microorganisms-13-01830-s001.zip › microorganisms-3781629-supplementary.pdf]

Supplementary Table S1: Genera detected from negative extraction (EC) and negative amplification (NTC) controls. Data are sequence counts.

Genus	EC1	EC2	EC3	EC4	EC5	EC6	EC7	NTC1	NTC2	NTC3	NTC4	NTC5	NTC6	NTC7
Acinetobacter	0	1	0	0	0	0	1	0	0	0	0	0	0	1
Unclassified *Actinobacteria*	0	2	0	0	0	0	1	1	1	0	0	1	2	1
Actinomyces	0	1	0	0	1	0	0	0	0	0	1	0	0	0
Alloprevotella	0	0	1	0	0	0	0	0	0	0	0	0	0	0
Unclassified *Alphaproteobacteria*	0	1	0	0	0	0	0	0	0	0	0	0	0	0
Anaerococcus	0	0	1	1	0	0	0	0	0	0	0	0	0	0
Anthococcus	0	0	0	0	0	0	0	1	0	0	0	0	0	0
Azomonas	0	0	0	0	0	0	0	0	0	0	1	0	0	0
Unclassified *Bacillaceae*	0	1	0	0	0	0	0	0	0	0	0	0	0	0
Unclassified *Bacillales*	2	3	0	1	0	1	0	0	4	0	0	0	0	1
Unclassified *Bacilli*	2	3	0	0	1	0	1	0	0	0	3	1	0	2
Bacillus	1	0	0	0	0	0	0	1	0	0	0	0	0	0
Unclassified *Bacteria*	2	12	0	1	0	0	3	3	4	2	1	2	2	2
Bergeyella	0	0	0	0	0	0	2	0	0	0	0	0	0	2
Unclassified *Betaproteobacteriales*	0	2	0	0	1	0	0	1	0	0	0	0	0	0
Unclassified *Bifidobacteriaceae*	0	0	0	0	0	0	0	0	0	0	0	0	0	1
Bifidobacterium	0	0	0	0	0	0	0	0	1	0	0	0	1	0
Bradyrhizobium	0	0	2	0	6	1	0	0	0	0	1	0	0	0
Burkholderia-Caballeronia-Paraburkholderia	8	0	0	3	3	2	0	1	1	1	1	2	2	0
Unclassified *Burkholderiaceae*	3	0	0	0	3	1	0	1	1	1	0	0	1	0
Unclassified *Carnobacteriaceae*	0	0	0	0	0	0	0	0	0	1	0	0	0	1
Unclassified *Caulobacteraceae*	0	1	0	0	0	0	0	0	0	0	0	0	0	0
Unclassified *Cellulomonadaceae*	1	0	0	0	0	0	0	0	0	0	0	0	0	0
Unclassified *Corynebacteriaceae*	0	0	2	0	0	0	0	0	0	0	0	1	0	0
Corynebacterium	0	1	0	1	0	0	0	0	0	0	0	1	1	0
Cutibacterium	3	1	1	0	1	0	0	3	4	0	3	0	1	0
Dolosigranulum	1	0	0	0	0	0	0	0	1	0	0	0	0	0
Dyadobacter	0	1	0	0	0	0	0	0	0	0	0	0	0	0
Endobacter	0	1	0	0	0	0	0	0	0	0	0	0	0	0
Enhydrobacter	0	0	0	0	0	0	0	1	0	1	0	0	0	0
Enterobacter	0	0	1	0	0	0	0	0	0	0	0	0	0	0

Unclassified *Enterobacteriaceae*	0	0	0	0	0	0	0	0	0	0	2	0	0	0
F0332	0	0	0	0	0	0	0	0	1	0	0	0	0	0
Fibrisoma	0	3	0	0	0	0	0	0	0	0	0	0	0	0
Flavisolibacter	0	3	0	0	0	0	0	0	0	0	0	0	0	0
Unclassified *Flavobacteriaceae*	0	2	0	0	0	0	0	0	0	0	0	0	0	0
Unclassified *Gammaproteobacteria*	0	1	0	0	0	0	0	0	0	0	0	0	0	0
Gemella	1	0	0	0	1	1	1	0	0	0	1	0	3	1
Granulicatella	1	1	0	0	0	1	2	0	2	1	1	1	0	2
Haemophilus	0	0	1	0	0	0	0	0	0	0	1	0	0	0
Halomonas	0	0	0	0	0	1	0	0	0	0	0	0	0	0
JGI_0000069-P22	0	0	0	0	0	0	0	0	0	0	0	0	0	1
Unclassified *Lactobacillales*	3	4	1	0	0	0	2	2	0	1	0	0	0	0
Lactobacillus	0	0	1	1	1	0	1	0	0	0	0	0	0	0
Leptotrichia	0	0	0	0	0	0	1	0	0	0	0	0	0	0
Unclassified *Leptotrichiaceae*	0	0	0	0	0	0	0	0	0	0	0	0	0	1
Mesorhizobium	0	0	0	1	0	0	0	0	0	0	0	0	0	0
Unclassified *Microbacteriaceae*	0	2	0	0	0	0	0	0	0	0	0	0	0	0
Unclassified *Micrococcaceae*	0	0	0	0	0	0	3	0	0	1	1	0	0	0
Unclassified *Micrococcales*	0	0	0	0	0	0	1	0	0	1	0	0	0	1
Unclassified *Myxococcales*	0	1	0	0	0	0	0	0	0	0	0	0	0	0
Neisseriaceae	0	0	0	0	0	0	0	0	0	0	0	0	0	1
Unclassified *Neisseriaceae*	0	0	0	0	0	1	1	0	0	0	0	0	0	0
Niveispirillum	0	1	0	0	0	0	0	0	0	0	0	0	0	0
Niveitalea	0	1	0	0	0	0	0	0	0	0	0	0	0	0
Oligotropha	0	0	1	0	0	0	0	0	0	0	0	1	0	0
Unclassified *Oxyphotobacteria*	0	1	0	0	0	0	0	0	0	0	0	0	0	0
Paracoccus	3	0	0	0	0	0	0	0	0	0	0	0	0	0
Unclassified *Pasteurellaceae*	0	1	0	0	0	0	0	0	1	0	0	0	0	1
Pelomonas	0	0	1	0	1	0	0	0	0	1	0	0	0	0
Peptoniphilus	0	0	0	0	0	1	0	0	0	0	0	0	0	0
Prevotella_7	0	0	0	0	0	0	0	0	0	0	0	0	0	1
Unclassified *Propionibacteriaceae*	0	3	0	1	1	0	0	0	1	0	0	0	1	0
Unclassified *Proteobacteria*	0	2	0	0	0	0	0	0	0	0	0	0	0	0
Unclassified *Pseudomonadaceae*	2	1	0	1	0	0	0	0	0	0	0	0	0	0
Pseudomonas	0	4	0	2	0	0	0	0	0	0	0	0	0	0
Psychrobacter	0	0	0	0	0	0	1	0	0	0	0	0	0	0

Ralstonia	15	4	2	6	5	3	0	1	1	1	0	1	0	0
Unclassified *Rhizobiaceae*	0	1	0	1	0	0	0	0	0	0	0	0	0	0
Unclassified *Rhodobacteraceae*	0	0	0	0	0	0	1	0	0	0	0	0	0	0
Rothia	1	1	2	1	1	0	2	1	1	2	0	1	1	9
Rubinisphaera	0	3	0	0	0	0	0	0	0	0	0	0	0	0
Saccharimonadaceae	0	1	0	0	0	0	0	0	0	0	0	0	0	0
Unclassified *Sphingomonadaceae*	0	1	0	0	1	0	0	0	0	0	0	0	0	0
Sphingomonas	0	0	0	0	2	0	0	0	0	0	0	0	0	0
Sporocytophaga	0	0	0	0	0	0	0	0	1	0	0	0	0	0
Unclassified *Staphylococcaceae*	1	0	0	0	1	0	0	0	0	0	0	0	0	0
Staphylococcus	4	2	3	2	1	2	0	4	3	1	3	2	1	1
Unclassified *Streptococcaceae*	1	2	0	1	0	0	1	1	0	0	0	0	0	2
Streptococcus	15	14	10	10	11	3	5	13	10	4	7	3	9	10
uncultured	0	2	0	0	0	1	0	0	0	0	0	0	0	0
Veillonella	2	2	1	1	0	0	1	1	1	0	0	0	0	0
Unclassified *Veillonellaceae*	0	1	1	1	0	0	1	0	0	0	1	0	0	0
Unclassified *Weeksellaceae*	2	0	0	0	0	0	0	0	0	0	0	0	0	0
Unclassified *Xanthobacteraceae*	0	0	0	0	1	0	0	0	0	1	0	0	0	0

Supplementary Table S2. Outputs of multivariate linear mixed effects models for the impact of maternal and infant characteristics on milk microbiota alpha diversity and composition. Time postpartum was included as an interaction for each variable. Variables and interactions with significant P-values and adjusted P-values are highlighted in bold.

Response	Variable	Estimate	Standard Error	P-value	Adjusted P-value
Shannon	Maternal age	0.043405765	0.015650658	**0.005791212**	**0.044399291**
	1 week	2.976082351	0.860414987	**0.000594387**	**0.007030747**
	2 months	0.326471552	0.715027523	0.648200147	0.813196549
	3 months	1.330210561	0.697008516	0.057002448	0.203985157
	4 months	1.261651773	0.758155042	0.096813643	0.26543608
	5 months	1.201196413	0.739196757	0.10489218	0.280163629
	6 months	0.887402057	0.740731557	0.231569289	0.454445465
	9 months	1.789240388	0.797812463	**0.025415411**	0.117246674
	12 months	0.051966024	0.808788711	0.948799012	0.979256037
	Maternal allergy	0.256580408	0.101559555	**0.013721596**	0.080010434
	Overweight or obese	0.220286829	0.095641656	**0.024603385**	0.117078176
	Autumn or winter birth	0.212136691	0.076225133	**0.006961312**	0.051364655
	Pet cats or dogs	-0.021440881	0.168590035	0.898861139	0.961572381
	Maternal age:1 week	-0.083469572	0.02424278	**0.000629416**	**0.007238286**
	Maternal age:2 months	-0.012691728	0.020998939	0.545897777	0.746198052
	Maternal age:3 months	-0.042654281	0.020147035	**0.034825154**	0.150183477
	Maternal age:4 months	-0.032911253	0.022080341	0.136811964	0.338919294
	Maternal age:5 months	-0.036018893	0.021326216	0.091952914	0.262541423
	Maternal age:6 months	-0.018893139	0.021553313	0.381201854	0.597793817
	Maternal age:9 months	-0.049455272	0.022847923	**0.030962867**	0.137834698
	Maternal age:12 months	0.000901746	0.023241707	0.969068596	0.9809154
	1 week:pet cats or dogs	-0.281892366	0.26262785	0.283702183	0.509890815
	2 months:pet cats or dogs	0.150842436	0.221956467	0.497120909	0.702416575

	3 months:pet cats or dogs	0.305160402	0.215858767	0.158181328	0.362847234
	4 months:pet cats or dogs	-0.045206378	0.226187313	0.841682486	0.933913104
	5 months:pet cats or dogs	-0.021882319	0.227138831	0.923295987	0.977607516
	6 months:pet cats or dogs	0.010650284	0.231262927	0.963289405	0.979256037
	9 months:pet cats or dogs	0.108493148	0.253796848	0.669238387	0.829535006
	12 months:pet cats or dogs	0.193384051	0.260467289	0.458208312	0.661155487
Richness	Maternal age	1.557986287	1.030851536	0.13171428	0.330483103
	1 week	260.7492499	53.25865823	**1.40505E-06**	**3.23161E-05**
	2 months	9.998180246	43.5613257	0.818581103	0.921592254
	3 months	62.51018581	42.29873675	0.140234262	0.343532452
	4 months	-3.969969185	46.02369429	0.931302746	0.979256037
	5 months	-27.62210264	44.48121076	0.534958765	0.740712136
	6 months	-44.90738248	45.1290102	0.32028418	0.541214901
	9 months	43.30754445	48.17075171	0.369158424	0.592370494
	12 months	-29.78676299	48.82578266	0.542158467	0.745693042
	Autumn or winter birth	9.843777431	9.584986809	0.305193285	0.530882437
	IAP use	-10.02045659	9.631929419	0.298976432	0.522262627
	Pet cats or dogs	-12.50203996	10.98318266	0.255883386	0.486619787
	Maternal age:1 week	-6.8487511	1.455626276	**3.46514E-06**	**7.55036E-05**
	Maternal age:2 months	-0.625873844	1.252468878	0.617548779	0.79646478
	Maternal age:3 months	-2.03075998	1.200375769	0.091461072	0.262541423
	Maternal age:4 months	-0.731344571	1.321491298	0.580276383	0.767522117
	Maternal age:5 months	0.351782414	1.282277605	0.783961174	0.896574381
	Maternal age:6 months	1.232835704	1.286056752	0.338320309	0.555811937
	Maternal age:9 months	-1.334736516	1.368445998	0.329954481	0.550760181
	Maternal age:12 months	0.842929658	1.395415282	0.546130458	0.746198052
	1 week:autumn or winter birth	1.015728644	13.00493326	0.937783876	0.979256037
	2 months:autumn or winter birth	-5.394882579	11.48108814	0.638683305	0.806142953
	3 months:autumn or winter birth	-3.987680338	11.23805994	0.722897215	0.855084134

	4 months:autumn or winter birth	13.74538786	12.08475858	0.256034768	0.486619787
	5 months:autumn or winter birth	4.437781007	12.10407616	0.714081096	0.851958425
	6 months:autumn or winter birth	44.87020302	12.06751704	**0.000228694**	**0.003155977**
	9 months:autumn or winter birth	-6.082579425	12.82784564	0.635631488	0.805713278
	12 months:autumn or winter birth	7.382608593	13.54426008	0.58599888	0.76773271
	1 week:IAP use	-9.916060753	13.19073375	0.452635446	0.659827727
	2 months:IAP use	7.213347026	11.57020848	0.533345491	0.740712136
	3 months:IAP use	10.03139853	11.32643541	0.376327754	0.594655305
	4 months:IAP use	11.94173165	11.93196102	0.317510875	0.540944454
	5 months:IAP use	4.359035356	12.00260709	0.716663742	0.852582728
	6 months:IAP use	1.879578944	11.97696211	0.875375849	0.943764587
	9 months:IAP use	2.530300254	12.61761716	0.841160267	0.933913104
	12 months:IAP use	5.689467486	13.56716706	0.675175043	0.831383221
	1 week:pet cats or dogs	-27.02701564	15.95207571	0.090971543	0.262541423
	2 months:pet cats or dogs	14.26384704	13.31677092	0.284752392	0.509890815
	3 months:pet cats or dogs	8.07357463	12.89976344	0.531755678	0.740712136
	4 months:pet cats or dogs	21.02691659	13.61587883	0.123296567	0.315091226
	5 months:pet cats or dogs	14.60030791	13.60132168	0.28370665	0.509890815
	6 months:pet cats or dogs	-10.14210831	13.82477403	0.46360282	0.666429054
	9 months:pet cats or dogs	22.43302145	15.2923978	0.143155872	0.346763111
	12 months:pet cats or dogs	9.088316748	15.72361709	0.563576617	0.756136862
Otu000001 *Streptococcus salivarius*	1 week	-3.881594494	1.355622989	**0.004385228**	**0.03574996**
	2 months	-0.592878722	1.125105376	0.598485755	0.779160699
	3 months	-1.532971079	1.061789521	0.149519093	0.355752326
	4 months	0.069504194	1.105067531	0.949877661	0.979256037
	5 months	0.924490962	1.107000858	0.404090793	0.624229807
	6 months	-0.313944495	1.165663275	0.787800968	0.898483748
	9 months	2.653082227	1.315229784	**0.044256589**	0.173095548
	12 months	-1.104434142	1.315192809	0.401485683	0.622528362

	Pet cats or dogs	0.50148359	1.037715782	0.629149829	0.802864133
	1 week:pet cats or dogs	3.251184097	1.612158652	**0.04431915**	0.173095548
	2 months:pet cats or dogs	-0.521082304	1.376534191	0.705204856	0.847226596
	3 months:pet cats or dogs	-0.101224344	1.33596909	0.939637749	0.979256037
	4 months:pet cats or dogs	-2.440037651	1.396661552	0.081318712	0.242625133
	5 months:pet cats or dogs	-2.55924526	1.399050846	0.068025531	0.219654574
	6 months:pet cats or dogs	0.085614157	1.434914292	0.952449015	0.979256037
	9 months:pet cats or dogs	-2.229741429	1.568895568	0.155938332	0.362145945
	12 months:pet cats or dogs	1.501389883	1.611281598	0.351932757	0.571373182
Otu000002 *Staphylococcus epidermidis*	1 week	0.198720218	0.641832473	0.756992327	0.876507713
	2 months	-1.658421408	0.566647379	**0.003595196**	**0.031008563**
	3 months	-1.616065396	0.560323266	**0.004109131**	**0.034717967**
	4 months	-1.970099934	0.594040104	**0.00098353**	**0.010440544**
	5 months	-1.855890789	0.589577007	**0.001750834**	**0.016856868**
	6 months	-2.440019444	0.591548824	**4.39958E-05**	**0.00082792**
	9 months	-3.37815215	0.633061197	**1.48279E-07**	**4.0925E-06**
	12 months	-2.459671645	0.671950845	**0.000280248**	**0.003742673**
Otu000005 *Ralstonia pickettii*	1 week	-1.043264934	0.994858884	0.294881073	0.519492614
	2 months	0.902872506	0.885068731	0.308217228	0.532114136
	3 months	1.489219012	0.869814918	0.08756542	0.258915154
	4 months	2.061574162	0.940563667	**0.028893289**	0.131205314
	5 months	2.113659543	0.943111003	**0.025488407**	0.117246674
	6 months	0.855277424	0.965698479	0.376263578	0.594655305
	9 months	-0.468431676	0.954073368	0.623672622	0.799612637
	12 months	-0.748993057	0.981072017	0.445585051	0.656484736
	Autumn or winter birth	-0.204560154	0.853427913	0.810667795	0.91949717
	1 week:autumn or winter birth	-0.206100501	1.303477262	0.874434042	0.943764587
	2 months:autumn or winter birth	-1.231844122	1.155518733	0.286967536	0.509890815
	3 months:autumn or winter birth	-0.779605812	1.141035906	0.494803584	0.702416575

	4 months:autumn or winter birth	-1.767366535	1.215797376	0.146724515	0.353162496
	5 months:autumn or winter birth	-4.148758986	1.210619685	**0.000665106**	**0.007246154**
	6 months:autumn or winter birth	-1.822101262	1.224826132	0.137532467	0.338919294
	9 months:autumn or winter birth	0.252329046	1.278916272	0.843679954	0.933913104
	12 months:autumn or winter birth	1.599823061	1.353264437	0.237727745	0.459903207
Otu000006 *Streptococcus lactarius*	1 week	-1.910386596	0.814058989	**0.019357128**	0.097729892
	2 months	1.371650018	0.718362454	0.056834693	0.203985157
	3 months	3.615555579	0.71030172	**5.24095E-07**	**1.27633E-05**
	4 months	4.033319793	0.753194124	**1.3549E-07**	**4.00663E-06**
	5 months	5.127522641	0.747596491	**2.24526E-11**	**9.29539E-10**
	6 months	5.425182342	0.750050584	**1.98777E-12**	**9.14374E-11**
	9 months	5.924521533	0.80287896	**7.42003E-13**	**3.83987E-11**
	12 months	3.205804318	0.852364726	**0.000190682**	**0.002784224**
Otu000007 *Burkholderia contaminans*	Maternal age	-0.26568506	0.111865932	**0.017965663**	0.0918245
	1 week	2.333713663	6.03753531	0.699291633	0.844042962
	2 months	-6.6202467	5.232616482	0.2065146	0.429611305
	3 months	-6.848679908	5.013733055	0.172689906	0.382318829
	4 months	-9.495878781	5.437122854	0.081461095	0.242625133
	5 months	-3.677796955	5.116891473	0.472698195	0.677152432
	6 months	3.703480384	5.378516414	0.491476626	0.701625252
	9 months	-10.6668702	5.593425248	0.057195946	0.203985157
	12 months	-8.842042638	5.630817936	0.117095769	0.302985303
	Parity two	3.451044548	1.302754151	**0.008352692**	0.057633578
	Parity three and more	2.483488656	1.346671348	0.065812201	0.217970011
	Overweight or obese	0.292912445	1.247192061	0.814426857	0.921236936
	Autumn or winter birth	-1.500745683	0.980720005	0.126650927	0.319755205
	Maternal age:1 week	-0.096399894	0.175427495	0.582940077	0.76773271
	Maternal age:2 months	0.26472104	0.158586388	0.095818021	0.265245748
	Maternal age:3 months	0.340790795	0.149157604	**0.022835696**	0.111223273

	Maternal age:4 months	0.443731324	0.163077536	**0.006779438**	0.051030682
	Maternal age:5 months	0.28799406	0.153259112	0.060930146	0.206762955
	Maternal age:6 months	-0.008338653	0.159598926	0.958356326	0.979256037
	Maternal age:9 months	0.497396719	0.167667111	**0.003182576**	**0.028033756**
	Maternal age:12 months	0.278769609	0.17205781	0.105929802	0.281121397
	1 week:parity two	0.424522096	2.06688658	0.837361965	0.933913104
	2 months:parity two	-1.722523029	1.782030659	0.334295895	0.551388448
	3 months:parity two	-2.772066083	1.726330303	0.109085809	0.287653024
	4 months:parity two	-5.9984215	1.876351351	**0.001493969**	**0.015085446**
	5 months:parity two	-6.47632559	1.832841938	**0.0004555**	**0.005893027**
	6 months:parity two	-3.668988844	1.83568628	**0.046285292**	0.175799184
	9 months:parity two	-6.939660171	1.977694229	**0.000497764**	**0.00624467**
	12 months:parity two	-1.750580052	2.174750746	0.421285833	0.631928749
	1 week:parity three and more	-1.361544816	2.214545426	0.538995288	0.743813497
	2 months:parity three and more	-0.728439288	1.860202571	0.695559262	0.844042962
	3 months:parity three and more	-3.319202072	1.791365723	0.064610077	0.215714288
	4 months:parity three and more	-3.940895959	1.934491728	**0.042259186**	0.17152258
	5 months:parity three and more	-3.978001702	1.934374572	**0.040351824**	0.167291285
	6 months:parity three and more	-2.493915032	1.956903131	0.2032176	0.424909527
	9 months:parity three and more	-6.044885788	2.137096931	**0.004895681**	**0.038977154**
	12 months:parity three and more	0.262057639	2.254472289	0.907517147	0.965840871
	1 week:overweight or obese	-4.99616104	1.976608535	**0.011845782**	0.073196324
	2 months:overweight or obese	0.082699404	1.708380571	0.961414616	0.979256037
	3 months:overweight or obese	-2.328145459	1.686105813	0.168100669	0.378091448
	4 months:overweight or obese	2.191102574	1.765844688	0.215375867	0.43495419
	5 months:overweight or obese	1.435023148	1.781815307	0.421066314	0.631928749
	6 months:overweight or obese	-1.407242467	1.770115509	0.427069037	0.638290907
	9 months:overweight or obese	-2.322684195	1.917613687	0.226482322	0.45078693
	12 months:overweight or obese	-4.255561859	1.974613424	**0.031712548**	0.13967016

	1 week:autumn or winter birth	0.966256008	1.517267187	0.524569385	0.733688261
	2 months:autumn or winter birth	0.607926121	1.316874681	0.644579903	0.811112705
	3 months:autumn or winter birth	-1.436975663	1.307744934	0.272485381	0.508148413
	4 months:autumn or winter birth	-0.581917849	1.392386911	0.676213833	0.831383221
	5 months:autumn or winter birth	-1.893248147	1.416674982	0.182133432	0.392725213
	6 months:autumn or winter birth	-1.566433922	1.411089283	0.267593417	0.501283596
	9 months:autumn or winter birth	1.408091302	1.47732647	0.341065221	0.558106726
	12 months:autumn or winter birth	3.73521989	1.541768289	**0.015821315**	0.087333661
Otu000008<br>*Rothia mucilaginosa*	1 week	-2.734220918	1.648976264	0.097980655	0.266003198
	2 months	2.600171495	1.369212395	0.058205971	0.203985157
	3 months	1.548793892	1.292938755	0.23161351	0.454445465
	4 months	3.429433856	1.345242939	**0.011131218**	0.069823092
	5 months	1.531744675	1.347425266	0.256239405	0.486619787
	6 months	0.964500725	1.418316514	0.496834167	0.702416575
	9 months	5.494232573	1.599597462	**0.000647155**	**0.007241137**
	12 months	2.270178305	1.599839379	0.15658001	0.362145945
	Overweight or obese	1.838347425	0.701893111	**0.01082382**	0.068939408
	Pet cats or dogs	0.39841345	1.254352254	0.750918205	0.875999768
	1 week:pet cats or dogs	2.70244478	1.961449419	0.168953908	0.378091448
	2 months:pet cats or dogs	-3.388148981	1.675658441	**0.043780077**	0.173095548
	3 months:pet cats or dogs	0.967748509	1.626738739	0.552218406	0.749568591
	4 months:pet cats or dogs	-1.855697527	1.700277311	0.275688692	0.50953178
	5 months:pet cats or dogs	1.647302916	1.702928224	0.333903869	0.551388448
	6 months:pet cats or dogs	3.073329328	1.746308748	0.079110709	0.240822307
	9 months:pet cats or dogs	-0.976470069	1.908686416	0.609185297	0.790604116
	12 months:pet cats or dogs	2.075557252	1.960148968	0.290217515	0.513461758
Otu000009<br>*Streptococcus parasanguinis*	1 week	0.205747255	0.899594539	0.819193115	0.921592254
	2 months	0.130185146	0.794526744	0.869919308	0.943764587
	3 months	1.247216159	0.785707104	0.113115616	0.296391551

	4 months	-0.689257696	0.832895781	0.408355815	0.626145582
	5 months	-0.552608688	0.826470316	0.504059376	0.709797896
	6 months	-0.677839637	0.8293429	0.414165303	0.630383954
	9 months	1.823738902	0.887351253	**0.040408523**	0.167291285
	12 months	2.290159196	0.941561334	**0.015374278**	0.086012855
	Caesarean birth	-1.443600658	0.711774659	**0.046005281**	0.175799184
Otu000010 *Gemella haemolysans*	1 week	0.7815158	0.91608554	0.394033533	0.615584463
	2 months	-2.132635049	0.811277625	**0.008858219**	0.060119714
	3 months	-1.600014603	0.802796326	**0.046849527**	0.176324583
	4 months	-1.615191137	0.850072574	0.058045827	0.203985157
	5 months	-0.909499847	0.842790192	0.281078432	0.509890815
	6 months	-0.647601161	0.845966461	0.444353656	0.656484736
	9 months	1.073577224	0.904722499	0.235969401	0.459903207
	12 months	0.428791181	0.958548142	0.654836737	0.816964819
	Overweight or obese	2.249674266	0.684091364	**0.00161998**	**0.015968376**
	IAP use	-0.259009931	0.545356431	0.63639672	0.805713278
Otu000011 *Acinetobacter johnsonii*	Maternal age	0.167925068	0.073077265	**0.022318399**	0.109997822
	1 week	8.056634789	3.337894506	**0.016212809**	0.087480762
	2 months	5.385905963	2.855329416	0.059950639	0.205120367
	3 months	0.4635879	2.754884784	0.866445683	0.943764587
	4 months	6.703069832	2.967356132	**0.024398802**	0.117078176
	5 months	4.775526245	2.863378028	0.096103532	0.265245748
	6 months	7.928796131	2.954446208	**0.007569037**	0.054027262
	9 months	7.607902462	3.093438289	**0.014318537**	0.082331585
	12 months	5.769703783	3.154500772	0.06809834	0.219654574
	Autumn or winter birth	1.821743645	0.680573269	**0.007858125**	0.055140062
	IAP use	-0.578483522	0.680861076	0.396255083	0.616727835
	Maternal age:1 week	-0.244424022	0.09785722	**0.01287345**	0.077240703
	Maternal age:2 months	-0.156152728	0.085093644	0.067202988	0.219654574

	Maternal age:3 months	-0.026833045	0.081140846	0.741039152	0.869094076
	Maternal age:4 months	-0.213380163	0.0886238	**0.016481883**	0.087480762
	Maternal age:5 months	-0.10792146	0.085880788	0.209581459	0.430230755
	Maternal age:6 months	-0.244313976	0.086885519	**0.005155135**	**0.040268415**
	Maternal age:9 months	-0.240978872	0.092809526	**0.009746598**	0.065082124
	Maternal age:12 months	-0.179073225	0.094445022	0.058633415	0.203985157
	1 week:autumn or winter birth	-1.685019718	0.875818765	0.055032095	0.201622011
	2 months:autumn or winter birth	0.916835446	0.775199823	0.237593396	0.459903207
	3 months:autumn or winter birth	-0.595016326	0.763849407	0.436438267	0.647618074
	4 months:autumn or winter birth	-1.685144527	0.817955721	**0.039995544**	0.167291285
	5 months:autumn or winter birth	-1.441299601	0.822116479	0.080301301	0.242625133
	6 months:autumn or winter birth	-1.371684136	0.821505051	0.095717986	0.265245748
	9 months:autumn or winter birth	-1.398783774	0.860433727	0.10476483	0.280163629
	12 months:autumn or winter birth	-1.729387768	0.91564509	0.059612538	0.205120367
	1 week:IAP use	0.977849222	0.879475415	0.266832008	0.501283596
	2 months:IAP use	-1.189541802	0.777263425	0.126666313	0.319755205
	3 months:IAP use	0.409695636	0.765334251	0.592716433	0.774083922
	4 months:IAP use	1.255646307	0.809496049	0.121620222	0.312737715
	5 months:IAP use	-1.190726274	0.811887855	0.143228241	0.346763111
	6 months:IAP use	0.864627071	0.810756883	0.286836124	0.509890815
	9 months:IAP use	0.559222865	0.856788414	0.514307009	0.721773226
	12 months:IAP use	0.173087803	0.917486895	0.850453965	0.9364041
Otu000012 *Enterobacter* sp.	1 week	2.244743976	0.784295481	**0.004403981**	**0.03574996**
	2 months	1.61222257	0.672554987	**0.016935284**	0.088749465
	3 months	1.111032872	0.66254603	0.094267739	0.265245748
	4 months	0.707302857	0.693705356	0.308471963	0.532114136
	5 months	0.876903123	0.694750679	0.207541693	0.429611305
	6 months	2.638808631	0.677702475	**0.000113871**	**0.001813173**
	9 months	1.392249462	0.766377914	0.069926786	0.222689919

	12 months	0.613926486	0.813777611	0.450987053	0.659747845
	Autumn or winter birth	1.041591169	0.441797224	**0.021093344**	0.105212584
	Infant male sex	1.128089988	0.78008502	0.148887525	0.355752326
	1 week:infant male sex	-3.530082929	1.128843562	**0.001877888**	**0.017669219**
	2 months:infant male sex	-1.433353756	0.998339233	0.151775889	0.357018285
	3 months:infant male sex	-0.344659728	0.987839675	0.727327028	0.857872904
	4 months:infant male sex	-0.453974376	1.050666252	0.66588827	0.827861092
	5 months:infant male sex	-1.268148876	1.040176668	0.223416809	0.446833617
	6 months:infant male sex	-2.309197608	1.055230211	**0.029156736**	0.131205314
	9 months:infant male sex	-2.35758986	1.112980214	**0.034694787**	0.150183477
	12 months:infant male sex	-1.42359011	1.181061841	0.228692036	0.45300719
Otu000014 *Veillonella* sp.	1 week	-2.401475916	1.358344399	0.077723263	0.239681416
	2 months	1.64711096	1.209425449	0.173905602	0.382962336
	3 months	0.377141784	1.18857893	0.751159222	0.875999768
	4 months	-0.396137864	1.284649787	0.757946284	0.876507713
	5 months	-0.372655594	1.288278399	0.772506527	0.890857109
	6 months	-1.332566211	1.318387267	0.31266097	0.534882817
	9 months	2.422449946	1.303065257	0.06365375	0.214249209
	12 months	0.52011585	1.339419101	0.697959816	0.844042962
	Maternal allergy	1.983975072	0.718548163	**0.007071945**	0.051364655
	Autumn or winter birth	2.973939466	1.160754318	**0.010697443**	0.068939408
	1 week:autumn or winter birth	-1.600686037	1.778998793	0.368706978	0.592370494
	2 months:autumn or winter birth	-2.883391952	1.578706583	0.068443092	0.219654574
	3 months:autumn or winter birth	-0.891271438	1.559486586	0.567934381	0.756136862
	4 months:autumn or winter birth	0.352622523	1.660442475	0.831915487	0.933368595
	5 months:autumn or winter birth	0.125532857	1.653945625	0.939532347	0.979256037
	6 months:autumn or winter birth	2.384151976	1.673008312	0.154815645	0.362111169
	9 months:autumn or winter birth	-4.349557936	1.746599983	**0.013111578**	0.077545616
	12 months:autumn or winter birth	-1.99882758	1.846939614	0.279700743	0.509890815

Otu000017 *Haemophilus parainfluenzae*	1 week	0.73272697	0.681519554	0.282860186	0.509890815
	2 months	-1.458106873	0.60464762	**0.016281537**	0.087480762
	3 months	-0.745703967	0.59829912	0.213268442	0.432809485
	4 months	-1.11777635	0.633157463	0.078156984	0.239681416
	5 months	-0.71327199	0.627389597	0.256170712	0.486619787
	6 months	-0.477228118	0.630096061	0.449202613	0.659467665
	9 months	0.129475395	0.673789121	0.847699694	0.935860462
	12 months	1.273420371	0.713483404	0.074927649	0.233233435
	Maternal allergy	1.975755644	0.503630173	**0.00019503**	**0.002784224**
	Autumn or winter birth	1.372430202	0.377304147	**0.00053099**	**0.006465587**
	IAP use	0.025595815	0.365260168	0.944367071	0.979256037
Otu000019 *Streptococcus anginosus*	Overweight or obese	-0.033776976	0.629277665	0.957329124	0.979256037
	Pet cats or dogs	0.181782127	0.586848614	0.757554355	0.876507713
Otu000020 *Granulicatella adiacens*	1 week	0.245192528	1.397404489	0.860793269	0.940286051
	2 months	0.050922969	1.161978453	0.965063921	0.979256037
	3 months	0.539550329	1.099446957	0.623852372	0.799612637
	4 months	-0.202185323	1.142696554	0.859639018	0.940286051
	5 months	1.895627476	1.144284251	0.09830553	0.266003198
	6 months	1.696778184	1.201688152	0.15863611	0.362847234
	9 months	5.803346385	1.354962346	**2.24373E-05**	**0.000464451**
	12 months	5.486658446	1.354914101	**6.0196E-05**	**0.00103838**
	Maternal allergy	1.267240407	0.507781793	**0.01482666**	0.084085444
	Pet cats or dogs	0.270555812	1.019397329	0.790805924	0.899433111
	1 week:pet cats or dogs	0.292483342	1.66263374	0.860438196	0.940286051
	2 months:pet cats or dogs	-0.224842608	1.422906178	0.874515665	0.943764587
	3 months:pet cats or dogs	0.140781604	1.38293284	0.918962655	0.975514203
	4 months:pet cats or dogs	1.302836193	1.443398678	0.367219472	0.592370494
	5 months:pet cats or dogs	0.013980809	1.445464199	0.992287152	0.995460277
	6 months:pet cats or dogs	3.3258965	1.481071197	**0.02521477**	0.117246674

	9 months:pet cats or dogs	0.933652931	1.617820392	0.564151782	0.756136862
	12 months:pet cats or dogs	2.084211009	1.660015029	0.209919354	0.430230755
